# Preliminary validation of a scale to measure patient perceived similarity to their navigator

**DOI:** 10.1186/s13104-015-1341-3

**Published:** 2015-08-29

**Authors:** Mechelle Sanders, Paul Winters, Kevin Fiscella

**Affiliations:** Department of Family Medicine Research, University of Rochester School of Medicine and Dentistry, 1381 South Ave, Rochester, NY 14620 USA; Department of Public Health Sciences, University of Rochester School of Medicine and Dentistry, Rochester, USA

**Keywords:** Community health aids psychometrics reproducibility of results

## Abstract

**Objective:**

A central premise in deployment of community health workers (CHW) is that CHWs share key characteristics with their patients. We sought to develop a scale to measure this construct called the Perceived Navigator Similarity (PNS) questionnaire.

**Methods:**

We adopted items from a similarly developed scale, patient perceived similarity to their physicians, and examined its psychometric properties among 51 patients who were navigated for cancer care by a CHW.

**Results:**

Principal component analysis revealed two main factors: personal and ethnic. The scale was associated with greater satisfaction with navigation (p < 0.005) and cancer care (p < 0.05).

**Conclusion:**

The PNS shows promise for further validation in larger samples assessing navigator-patient similarity from the patient perspective.

**Electronic supplementary material:**

The online version of this article (doi:10.1186/s13104-015-1341-3) contains supplementary material, which is available to authorized users.

## Background

Community health workers (CHWs) are often utilized to help eliminate patient barriers, and improve access to care among the poor and underserved [1, 2]. One important role for CHWs is navigation of patients with suspected or known cancer [3]. Navigation involves assisting patients in obtaining care and services in addition to providing education and emotional support [3, 4].

An underlying premise behind use of CHWs is that the CHW shares common characteristics (i.e. commonality) with the clients or in the case of navigators, the patients they serve [5–7]. These characteristics often include race, ethnicity, language, culture, and community of residence. Potentially, these commonalities foster cultural competency and the ability to effectively engage patients [6, 8]. To date, there is no measure for assessing the extent to which patients perceive their navigator-CHW as similar to themselves.

Findings from physician to patient relationships suggest that patients report greater trust, satisfaction and adherence when they experience a sense of partnership with their physician [5, 7, 9]. Some data suggest that racial concordance improves partnership [10–12]. However, other data suggest that partnership is also driven by a personal connection that may transcend race or ethnicity [8, 13]. This personal connection between people is not unique to patient–physician relationships, but rather represents a fundamental aspect of social cognition [14]. It is this personal connection that represents a key element of the peer support provided by CHWs [15].

Based on the notion that human relationships are driven by deeper emotional connections, Street et al. [13] developed the perceived similarity scale, where patients rate the ways they perceive themselves as similar to their physicians. However, there is no comparable scale that assesses patients’ perceived similarity to their CHW.

The primary aim of this study was to address that gap, by developing and evaluating a measure that can be used to assess patient perceived commonality between patients/clients and their CHWs who served as navigators. We refer to it as the Perceived Navigator Similarity (PNS) scale.

We hypothesized that PNS scale would be correlated with patient satisfaction with navigation and patient satisfaction with cancer care. Specifically, we expected that patients perceiving themselves as more similar to their navigators would report improved satisfaction with their navigators and also report a more favorable experience of cancer care.

## Methods

### Description of parent study

We examined patient perceptions of CHWs who functioned as patient navigators, as part of the Patient Navigation Research Program (PNRP). The Program is sponsored by the National Cancer Institute, Center to Reduce Cancer Health Disparities. The PNRP is a nine site cooperative study, designed to rigorously evaluate the impact of patient navigation on receipt of diagnostic testing and treatment for patients with cancer screening abnormalities and/or diagnosed cancer [4].

Our site (Rochester, NY, USA) focused on evaluation of patient navigation, provided by CHWs, for those recently diagnosed with breast or colorectal cancer. Following informed consent, we randomly assigned participants to navigation or usual care. We surveyed participants at baseline, 3, 6, 9 and 12 months or study completion [16].

### Description of navigator training

The four navigators were CHWs (i.e. health promotion personnel). All had experience working in various community health organizations. Two were African American non-Hispanic, one was Hispanic (Spanish speaking), and one was White non-Hispanic. Their education levels varied from HS graduates to college graduates. The mean age was 40 years.

As a part of their initial training, navigators received intensive training from Cornell Empowering Families Project. The curriculum consisted of ten modules related to empowerment, communication skills, cultural competency, and assessing patient needs. In addition, the navigators received ongoing training on breast and colorectal cancer treatment, communication, and confidentiality [17, 18].

### Participant inclusion criterion

Study participants were recruited by research assistants (RA), from cancer treatment centers in Rochester, NY, USA. In order to be eligible for the study participants were required to be at least 18 years of age, recently diagnosed (<3 months) with breast or colorectal cancer, and could not have been working with any other cancer navigator or case manager for cancer. In addition, they could not be incarcerated, living in a nursing home, pregnant or have had a previous cancer diagnosis within 5 years. The study was approved by the institutional review board at the University of Rochester, and informed consent was obtained from all participants.

### Development of the measure

We adapted items from Street et al.’s similarities measure where patients rated their perceived similarity to their physician [13]. The scale consists of ten items: five related to personal similarity and five to ethnic similarity. Patients’ responded to each of these items using a 6 point Likert Scale (0, very different to 5, very similar). A total score was calculated by adding all items, with higher scores indicating greater perceived similarity.

We adapted Street et al.’s scale to our setting by substituting the words “my navigator” in place of “my doctor” in order to assess patient perceived similarity to their navigator. RA’s were instructed to take notes on any issues subjects may have had with completing the PSN. After ten subjects completed the measure; the team reviewed all subject comments and through consensus decided to keep all items on the scale. The adapted scale, PNS is shown in Additional file 1: Table S1.

### Participants

We developed the PNS near the end of the PNRP study; as a result the PNS was only administered to 51 of the 166 participants currently undergoing the intervention and assigned to navigation. Our sample included 42 breast cancer patients and 9 colorectal cancer patients resulting in a sample that was 90 % female. Ages ranged from 24 to 80 years, with mean of 55 years. Characteristics of the sample were comparable to that of the larger study (see Additional file 1: Table S2).

### Data collection and measures

Research assistants administered surveys to participants. When required, the research assistant read the questions to patients. Participants also provided demographic information related to their sex, age, race, ethnicity, marital status, income, health insurance status, highest grade completed in school, and employment. Surveys included Satisfaction with Navigator-Interpersonal dimension [17] and the Patient Satisfaction with Cancer Related Care [19]. The former assesses patients’ satisfaction with the interpersonal dimension of patient navigation. It includes items such as: “My navigator is dependable”, “My navigator cares about me personally” and “My navigator is easy to talk to”. The latter assesses patient satisfaction with cancer related care (both diagnostic and treatment services). It includes questions such “I felt that my health concerns were understood”, “I felt confident in how I dealt with the health care system”, and “I was satisfied with the care I received”. We anticipated moderate correlations with both these scales. Specifically, we hypothesized that perceived similarity with navigators, would be associated with greater satisfaction with overall cancer care and with satisfaction with the interpersonal dimensions of navigation.

### Analysis

To determine factor structure or dimensionality, we conducted a principal component analysis. Internal consistency reliability was assessed based on Cronbach’s alpha for the final scale items. All analyses were performed with SAS statistical packages. To assess construct validity, we assessed the correlation of the scale with patient Satisfaction with Navigator-Interpersonal and Satisfaction with Cancer Related Care scales. We hypothesized moderate correlations with these scales.

## Results

### Participant characteristics

Our final sample was based on responses from 51 participants. Participants did not differ from the non-participants in the intervention arm in terms of age, race, and gender or cancer type (Additional file 1: Table S2). Roughly 90 % of our sample was female, and 10 % male. They ranged from age 24–80, with the average age being 55. The participants reported an average income range of $30,000–$39,999, 93 % of our sample reported having a high school diploma or greater, and the average REALM-S score was 20.0 (indicating at least high school reading proficiency).

### Factor structure

Given the high rate of non-response to the free time question (64.7 %) and the spiritual beliefs question (58.8 %), these two items were dropped. The exploratory factor analysis results showed 2 factors with an eigenvalue greater than 1.0 (see Additional file 1: Table S3). Review of a scree plot displayed two dominant factors explaining 73 % of the variance. Based on the clustering of the factor loadings, we named the factors communication similarity (speak, reason, values, and communicate) and ethnic similarity (ethnicity, culture, race, and skin color).

### Reliability

The scale showed good internal consistency (Cronbach’s coefficient alpha = 0.77). The perceived similarity in communication behavior and perceived similarity in ethnicity subscales had Cronbach’s alphas of 0.79 and 0.93, respectively.

### Validity

As hypothesized, there was a modest positive correlation between the PNS and the 9-item Interpersonal dimension of satisfaction with patient navigation (r = 0.47, p value = 0.004), and with patient satisfaction with cancer related care (r = 0.35, p value = 0.02). As expected, the scale had no appreciable correlations with age, gender or education.

## Discussion

In this paper, we describe the development and preliminary validation of a brief scale designed to measure patients’ perceived similarity with their navigator. We observed two major factors: perceived similarity in communication behavior and perceived similarity in ethnicity. The scale was reliable showing reasonable internal consistency. The items showed reasonable face validity. Construct validity was shown through correlations with related constructs. Although our findings need to be replicated in a larger sample, additional measures of reliability (e.g. test–retest), and validity (prediction of patient outcomes); our preliminary findings, if replicated, offer promise for a simple way to measure concordance between patients and their navigators.

Our preliminary findings are similar to those of Street et al. [13]. Our findings suggest that the paradigms that patients use to assess similarity with their navigator are more complex than simply race and ethnicity. Our results suggest both interpersonal characteristics; as well as perceived racial and ethnic similarity are important to patients being navigated. The salience of relational factors is consistent with our qualitative findings [16] and with findings that human relationships represent a fundamental element of social cognition [14]. Our findings are also consistent with the patient-physician literature that suggests that physician communication is associated with higher levels of trust [20], and with the CHW literature that underscores salience of social connection [21].

Our findings are best interpreted in the context of the study limitations. We did not develop our scale de novo based on qualitative data from patients, but instead adapted items from an existing scale used for physicians. Two items (perceived use of free time and spirituality) from the original scale were dropped due to high rates of patient non-response. We suspect that non-response to these items reflects patients’ lack of perceived patient knowledge regarding these aspects of navigator’s lives. It is not uncommon for primary care physicians to address patients’ spiritual needs [22–24] and share aspects of their personal lives including hobbies, however appropriate [25]. Unlike relationships with physicians that may span many years, patient navigator relationships in this study were limited to maximum of 12 months and varied in intensity of contact. In other settings, CHWs relationships with clients may last longer. In this study CHWs may not have conversed with patients on spiritual needs and therefore the scale did not capture this aspect of commonality [26]. In addition, our sample was based exclusively on breast and colorectal cancer patients who had been assigned to the navigation arm of a randomized trial. Further study is needed to replicate our findings in larger, more diverse samples. We did not conduct test re-test reliability so we cannot comment on the stability of the measure over time. Last, further study is needed to determine whether this scale predicts patient outcomes [27]. If our findings are replicated, this scale could provide a measure for researchers to explore factors related to navigator effectiveness. It is plausible that perceived similarity will be associated not only with improved client satisfaction but also with improved client trust and potentially client’s perceptions of social support.

In conclusion, we present preliminary validation the PNS. While this scale requires validation in a larger and more diverse sample, it offers promise for assessing a key component of navigation provided by CHWs—patients’ perceived similarity to the navigator. This scale could prove useful in research related to CHW as well as program evaluation.

## Additional file

**Additional file 1.** Adapted perceived navigator similarity scale.
