# Supplementary material for: Preliminary validation of a scale to measure patient perceived similarity to their navigator
Source: BMC Res Notes. 2015 Aug 29;8:388. doi: 10.1186/s13104-015-1341-3 (PMC4552994; doi:10.1186/s13104-015-1341-3)
Supplement: Supplementary file 1 — Additional file 1. Adapted perceived navigator similarity scale. [file 13104_2015_1341_MOESM1_ESM.docx]

| **Table 1**. Perceived Navigator Similarity | |
| --- | --- |
| **Please indicate how different or how similar you and your Navigator are.** | |
| 1 | The way my navigator and I speak is… |
| 2 | The way my navigator and I reason about problems is… |
| 3 | My navigator and I have…styles of communication |
| 4 | My navigator and I have…general values in life |
| 5 | My navigator and I have…spiritual beliefs* |
| 6 | My navigator and I have…ethnic backgrounds |
| 7 | The types of people I spend my free time with and the types of people my navigator spends his/her free time with are…* |
| 8 | My navigator and I are…in terms of race |
| 9 | My navigator and I are…in terms of culture |
| 10 | My navigator and I are…in terms of skin color |

| **Table 2**. Comparison of demographic characteristics between the overall and validation samples | | | |
| --- | --- | --- | --- |
|  | **Entire Navigated Sample**  **(N= 168)** |  | **Sub-sample who completed the PNS**  **(N=51)** |
| **Subject Characteristics** | **%** |  | **%** |
| **Age** |  |  |  |
| 20-39 | 9% |  | 10% |
| 40-49 | 23% |  | 23% |
| 50-59 | 30% |  | 27% |
| 60-69 | 28% |  | 29% |
| 70+ | 11% |  | 10% |
| **Sex** |  |  |  |
| Female | 90% |  | 90% |
| **Race/Ethnicity** |  |  |  |
| Non Hispanic-Afr American | 23% |  | 22% |
| Non Hispanic- White | 63% |  | 71% |
| Other | 14.7% |  | 8% |
| **Income** |  |  |  |
| Less than $10K | 14% |  | 12% |
| $10K-$19999 | 14% |  | 12% |
| $20k-$29999 | 12% |  | 8% |
| $30K-$39999 | 8% |  | 12% |
| $40K-$49999 | 4% |  | 0 |
| $50k or more | 30% |  | 37% |
| Missing | 18% |  | 20% |
| **Cancer Type** |  |  |  |
| Breast | 85 % |  | 82% |
| Colorectal | 15.9% |  | 18% |
| **Education** |  |  |  |
| Less than HS | 17% |  | 8% |
| HS/GED | 21% |  | 20% |
| More than HS | 62% |  | 73% |

| **Table 3**. Factors | | | | | |
| --- | --- | --- | --- | --- | --- |
|  | | Factor1  Λ=  3.04 |  | Factor2  Λ= 2.81 |  |
| PNS1 | 1. The way my navigator and I speak is... | 23 |  | 89 | * |
| PNS2 | 2. The way my navigator and I reason about problems is... | 26 |  | 81 | * |
| PNS3 | 3. My navigator and I have... styles of communication | 21 |  | 81 | * |
| PNS4 | 4. My navigator and I have... general values in life. | 5 |  | 74 | * |
| PNS6 | 6. My navigator and I have... ethnic backgrounds. | 86 | * | -13 |  |
| PNS8 | 8. My navigator and I are... in terms of race. | 89 | * | -24 |  |
| PNS9 | 9. My navigator and I are... in terms of culture | 78 | * | -3 |  |
| PNS10 | 10. My navigator and I are... in terms of skin color | 86 | * | -31 | * |
| Printed values are multiplied by 100 and rounded to the nearest integer. Values greater than 0.3 are flagged by an '*'.  Items 5 and 7 were not used in the analysis. | | | | | |
